# Blended learning in physical education: application and motivation

**DOI:** 10.3389/fpsyg.2024.1380041

**Published:** 2024-08-27

**Authors:** Dagmar Hrušová, David Chaloupský, Pavlína Chaloupská, Petr Hruša

**Affiliations:** Department of Recreology and Tourism, Faculty of Informatics and Management, University of Hradec Králové, Hradec Králové, Czechia

**Keywords:** physical education, motivation, university students, learning methods, blended learning, outdoor aerobic sport activities, wearables, Strava app

## Abstract

**Background:**

The aim of the research was to evaluate outdoor aerobic sport activities (OASA) in the physical education (PE) of university students using wearables and their potential to personalize the learning process and enhance motivation.

**Methods:**

In total, 368 university students participated. The OASA structure and the key points of application in PE were described. Descriptive statistics of the training units (*n* = 3,680) were processed. The students recorded their training data in the Strava app (10 sessions per semester), and the data were shared in the online sport community created on the Strava platform. Motivation was evaluated using a questionnaire. The focus was both on intrinsic motivation and extrinsic “ICT” motivation, based on Strava app features and tools.

**Results:**

The most preferred outdoor aerobic sport activities were running (58%), cycling (13%), and walking (16%). The results provided insight into motivation and performance analysis. Students’ motivation to participate in OASA was mainly in health concerns, such as staying in shape (94%), staying healthy (90%), and psychological concerns, such as having fun (88%), improving state of mind (88%), or relieving stress (83%). In achievement concerns, the motivation was a personal challenge (72%), while competing with others was ranked lowest (32%). The Strava app was a motivating tool for students to record, monitor, and analyze their individual activities and feel “connectedness” to the online sport community. 70% of students were motivated by the non-competitive character of PE, which gave them a personalized opportunity to train without being compared to others.

**Discussion:**

The OASA management, with the use of blended learning methods and the Strava app, uses a motivational approach to create, support, and maintain students’ healthy habits of physical activity through PE lessons. The need for students to be motivated to exercise can be confirmed in the analysis of the statistical descriptive parameters of running, cycling, and walking. There was a tendency for students to complete only the minimum required distance/time (not more). On the other hand, students enjoyed the training, and 99% of students confirmed that they would enroll again. That fact underlined the importance of motivating students with an effective learning strategy and giving support and guidance.

## 1 Introduction

Research focuses on the physical activity of university students. The authors emphasize that regular, targeted, adequate, and variable exercise and physical activity are an important part of university study as a primary prevention to compensate for sedentary study load and to develop fitness and motivation. Today’s overall decline in physical activity (PA) and increase in sedentarism through the periods of childhood, puberty, adolescence, and young adulthood is quite alarming (Llorente-Cantarero et al., 2020; McFadden, 2021; Podstawski et al., 2021; Pereira et al., 2022). The analysis of Guthold et al. (2020), based on 1.6 million school-going adolescents, estimated levels of insufficient PA across 146 countries to assess global, regional, and country-time trends in insufficient PA, showing that the majority of adolescents did not meet PA guidelines, putting their current and future health at risk. Decline of PA in childhood and adolescence can be seen in reduced participation in non-organized activities like active play and informal leisure-time sports (Kemp et al., 2021) and is influenced by the factors of environmental changes, especially in girls (Sember et al., 2020). In high school and university students, the main barriers were identified as lack of time, motivation, and accessible places (Silva et al., 2022). Kolář (2021) describes the importance of positive emotional relationship to PA and the formation of interpersonal and social bonds in conjunction with PA, which must first be established, so that later, in adulthood, the imprints of physical activity can emerge from the memory traces and bring with them similar positive emotional experiences. Later in life, they are more difficult to adopt. In a broader context, it is recommended that education administrators, clinicians, public health education funders, and families work together to motivate students to adopt behavioral and environmental changes for sustainable lifelong physical fitness and a healthy lifestyle (Bebeley et al., 2018; Ahmad et al., 2021).

There is a noticeable trend in current research to examine physical activity as a factor influencing quality of life, happiness, life satisfaction, and overall health (Zhang and Chen, 2019; An et al., 2020; Marquez et al., 2020; Posadzki et al., 2020). Practicing regular, moderate-to-vigorous PA in adolescence and young adulthood seems to have a positive effect on the level of their PA later in life (Tammelin et al., 2003; Pheiffer and Wierenga, 2019; Memon et al., 2021). From the opposite perspective, insufficient physical activity increases the risk of non-communicable diseases, poor physical and cognitive function, weight gain, and mental ill-health (Strain et al., 2024). Sedentary behavior has been shown to be strongly related to depression, anxiety, stress, and overall well-being (Thievel et al., 2021). The importance of adequate PA has also been recognized by the European Commission and Member States and highlighted as a priority in the Health-Enhancing Physical Activity (HEPA) project (European Commission, 2024). There is an evidence-based consensus on the recommendation of PA for health benefits across different guidelines to prevent hypertension, cardiovascular disease, cancer, and mental health disorders, with a widely accepted proposed minimum of 150 min of moderate aerobic activity or 75 min of vigorous activity a week; however, many adults and children do not achieve the recommended minimum (Khanji et al., 2022).

Outdoor sports such as running, cycling, or walking seem to be a good choice for adolescents. Possibly the most widespread individual outdoor physical activity is running, which has become popular and is considered a social phenomenon due to its ease and low cost (Rozmiarek et al., 2022). Benefits associated with social impacts of being in nature when performing outdoor sports have been clustered around physical health, mental health and well-being, education and lifelong learning, active citizenship, crime reduction, and anti-social behavior (Eigenschenk et al., 2019). Exercise in a natural environment can also promote focused attention and social interactions, which can have a positive impact on future intentions to exercise (Rogerson et al., 2016). The natural landscape can also play a role in perceived joy or calm (Paraskevopoulou et al., 2022). PA performed outdoors in natural environments is more beneficial for a range of psychological outcomes compared with urban environments, with large or moderate effects for anxiety, fatigue, positive affect and vigor, and a small effect for depression (Wicks et al., 2022). However, in terms of comparison of added health or behavioral benefits of outdoor sport versus indoor exercise, there is limited evidence (Noseworthy et al., 2023). In outdoor sport activities, the use of fitness tracking devices, applications, and networks has increased significantly over the last few years, and it is important to understand how they can influence users’ motivation through other factors such as social support, self-efficacy, or enjoyment (Melton et al., 2015; Hamid et al., 2019; Rivers, 2020).

Digitization of physical activity enables athletes to plan their physical activities (goal setting, quantification and recording of activity, real-time monitoring), share data on each session, track the performance of others, compare their performance, compete, receive incentives and rewards, and give and receive feedback (Soulé et al., 2021). There is a stronger positive effect of fitness tracker use on perceived physical health and psychological well-being (e.g., positive emotions, experienced meaningfulness of life, and sense of accomplishment) when fitness trackers are accompanied by mobile apps (Jin et al., 2022). Popular fitness tracking apps include, for example, Strava, Endomondo, Fitbit, Runkeeper, Runtastic, or MapMyRun. The shift toward fitness apps and activity trackers can be seen together with a change in behavior and a certain trend that people are increasingly adopting new digital sports solutions (Ruth et al., 2022). Runners who do not consider running as their main sport are more likely to use apps than those who consider running as their most important (or only) sport, while the training frequency has no significant contribution to the usage of apps (Janssen et al., 2020). The age of the athlete can also play a role in the use of sports apps and wearables. Users aged 24–29 were less interested in recording their sports training than those aged 30–49, when the latter group preferred wristbands or smartwatches, while smartphones remained the most used device among the younger (Soulé et al., 2021). The role of smartphone fitness tracking apps has been studied as a possible tool to increase motivation for physical activity at school PE (Adamčák et al., 2021).

The theoretical background of motivation in sport activities has been concerned with self-determination theory (SDT), which describes the tendency of people to strive for personal growth and the conditions that support their development (Jones et al., 2022). SDT distinguishes types of motivation, such as mental health and well-being, effective performance, creative problem-solving, and conceptual learning (Deci and Ryan, 2008; Stragier et al., 2018). SDT’s applications in education focus on facilitating the satisfaction of the basic psychological needs of both students and teachers (Ryan and Deci, 2020). In school PE, teachers should facilitate motivation to know something, to achieve something, or to experience stimulation. Intrinsic motivation is a powerful mechanism for encouraging sport participation and enjoyment in all age categories. Given its importance, the ability to measure intrinsic motivation is paramount for researchers and sport practitioners (Schüler et al., 2023). The evidence regarding self-determination theory has been analyzed in a systematic review within the school PE context, finding that autonomy, competence, and relatedness satisfactions were strongly correlated with autonomous student motivation and less strongly, but still positively, correlated with introjected regulation. These findings further revealed that relatedness in PE is associated with both peer and teacher influences (Vasconcellos et al., 2020). For the needs of practice, the main categories of running motivations are based on MOMS (Motivations of Marathoners Scales), with the following subcategories: physical motivation, social motivation, achievement motivation/life meaning, and psychological motivation (Ogles and Masters, 2003). It is important to consider goals because achieving them leads to a sense of well-being and behavioral compliance. For adolescents, goals such as contact and feeling challenged are paramount, while for young adults, health, figure/appearance, and distraction/catharsis predominate (Gut et al., 2022). Extrinsic sport motivation refers to a variety of actions and behaviors as a means to achieve goals (Wang et al., 2022). By understanding the links between motivation and processes, technology and app designers can better create engaging experiences with motivating features to encourage users to adopt healthy habits for physical activity (Oc and Plangger, 2022).

The potential of health and fitness-related technology for motivation is highlighted in this research, with a focus on feedback and goal support and exploring new modalities to support the contextual users’ needs (Rapp and Tirabeni, 2020). Acceptance of sport-related technology depends on simplicity of use and perceived usefulness, and users’ positive attitudes toward technologies can be significantly promoted by intrinsic sport motivation (Wang et al., 2022). In terms of social interactions, current solutions emphasize individual achievement and challenge while undervaluing the potential of positive social interactions such as collaborative challenge, which can be described as a desire to “achieve together” rather than “fight each other” (Ruth et al., 2022). The social elements in self-tracking technology contribute to the satisfaction of needs for both competence and connectedness, and meeting these needs leads to higher levels of autonomous motivation and improved well-being (Jones et al., 2022). Running-related technology attempts to meet the different needs of individual athletes through research into different types of runners, their attitudes, interests, and opinions, and how they differ in the use of technology (Janssen et al., 2020). The main outcomes of fitness tracking behavior lie in task motivation, task experience, PA level, and well-being/health (Jin et al., 2022). In professional athletes, running-related technologies are seen more as a convenient tool to facilitate the management of their sports training, as they possibly do not need high external motivation from technological support (Karahanoğlu et al., 2021). Amateur athletes need to be well motivated with respect to their individual needs and goal setting, similar to university or college students, who may perceive various benefits from fitness tracking apps and wearables use (Hamid et al., 2019; Ahmed and Leung, 2021; Ho et al., 2022; Rana et al., 2023; Russell et al., 2023). Wearables are recommended to facilitate a change in exercise behavior (Hewitt et al., 2022), perhaps most effective in sedentary or underactive adults (Nuss et al., 2021). Motivations for the use of wearables among college students have been examined; however, despite the positive impact of fitness trackers, students still did not meet the recommended physical guidelines (Wallar, 2021). This indicates a need for practice guidelines and tailored strategies in college PE.

There is an evident drive for curriculum innovation with a greater focus on PE (Blocker and Wahl-Alexander, 2018; Wen, 2023), suggesting that sport education in universities can positively impact students’ engagement through features like team affiliation, formal competition, and record-keeping (Choi et al., 2021). The significance of long-term physical exercise effect and the concept of lifelong PA are highlighted (Shi, 2023). The study by O’Brien et al. (2022) assessed the physical fitness levels of a sample of Canadian university students with a similar cohort over a 25- to 30-year time-lapse and found that physical fitness has decreased over the past 30 years, while sedentary time has increased. This suggests that even the students with knowledge of physical fitness theory and practice appeared to follow the same declining health pattern as the general population. The current trend toward an increasing sedentary lifestyle was indicated in the study of Czech university students (Šipl and Hurych, 2022), which found that 55% of participants were minimally active, with an average sitting time of more than 5.5 h a day. The study also showed that lower levels of PA are associated with higher values of total body fat in university students. Similarly, university students who have a sitting time of more than 7 h a day had a significantly lower fitness level in the study of Yoo et al. (2020). While most Mumbai University students stated that exercise is important to them, they still failed to work out for sufficient hours a week (Jambusaria et al., 2020). The state and status of PE in higher education were analyzed in the selected European countries in 66 tertiary institutions, suggesting that the transition to higher education may have a negative impact on students’ PA behavior and motivation (Podstawski et al., 2021). The authors found that PE is part of the curriculum in 68% of universities, bringing a more detailed analysis that examines the problems of insufficient PA levels and recommends that PE curriculum be strategically planned and implemented with improved funding (Podstawski et al., 2021).

Recommendations on innovation of college and university PE consider the use of modern methods, such as blended learning, that combines face-to-face and online learning teaching. The challenge is to integrate the benefits of the chosen learning activities to identify a desirable blend, with conceptualization describing to what extent a course is conducted face-to-face in online settings (Hrastinski, 2019). The systematic review of Wang et al. (2023) indicates that, despite the growing interest since 2018, blended learning in PE is in the initial stages of development. Several attempts of blended learning application in PE practice have been made, but the number of high-quality studies is limited. They imply that teachers need more training to improve their course design and management of online classes, including the use of multiple technologies as instructional support tools and the design of learning activities through various strategies (Wang et al., 2023). Teachers’ main challenge can be found in their unwillingness and negative perception of using technology for instruction, while educational institutions find it difficult to provide the correct and sufficient technological infrastructure as well as effective training support to their teachers (Rasheed et al., 2020).

Despite mixed results, there are some studies giving examples of practical usability of blended learning in PE. Based on students’ feedback on mobile flipped learning in a PE badminton course, it was verified that instructing students to identify the crucial concepts, carry out peer interaction, observe their practice videos, and provide the criteria for objective analysis could effectively support students’ self-reflection and enhance their learning performance (Lin et al., 2021). A blended learning approach was implemented in PE to teach dance-related classes through digital instruction to dance curricula, combining online and face-to-face classes, with improved effects on learning and increase of students’ satisfaction (Chao et al., 2021). A blended learning model helped to personalize learning process, increase university students’ participation in PE fitness running courses, and reduce dropout at the end of the course (Chaloupský et al., 2020). Teachers should facilitate students’ motivation through blended learning, as the digital support affects students’ perceived autonomy, competence, and relatedness, resulting in stronger engagement (Chiu, 2021). Sports practice has changed in recent years due to the limitations caused by the COVID-19 pandemic, distant learning, and the need to integrate technologies (Alvarez et al., 2022). YouTube as a video app appeared to be a “learning companion” for sports science students during COVID-19 to better understand the course content taught; however, distant learning also has its limitations, and there were differences in effectiveness between online instruction and classroom instruction, with the latter appearing to be more effective (Trabelsi et al., 2022). Some teachers reported difficulties in motivating students in distant learning (Moustakas and Robrade, 2022). Wang et al. (2023) identified major challenges of blended learning in instructional design, technological literacy and competency, self-regulation challenges, alienation and isolation, and belief challenges. Creating a habit of regular PA is one of the most important issues to focus on in education, as research has shown an alarming overall decline in physical activity associated with an increase in sedentary time per day among children and adolescents and also an increase in screen time per day (Thievel et al., 2021).

Regular physical activity is considered an efficient method of primary prevention, especially for students of technical studies, to balance sedentary time. A combination of fitness tracking apps and wearables in university PE, as approached in blended learning, may possibly help in searching for a solution. The research draws on a long-term experience and two previous research studies carried out by the authors (Chaloupský et al., 2019, 2020), investigating wearables in PE and their potential to increase students’ motivation to run, where we designed, implemented, and evaluated an innovative blended learning model (BLM) of running classes with the use of fitness trackers in smartphones. According to the best of our knowledge, the aim of this research is to evaluate outdoor aerobic sport activities (OASA) in PE of university students in the study curriculum at the Faculty of Informatics and Management at the University of Hradec Kralove (FIM UHK), Czech Republic, using wearables with the Strava app and their potential to effectively promote learning process, personalize methods, and enhance motivation in agile-blended learning. The research focus is to analyze students’ intrinsic motivation to participate in OASA, evaluate features and tools of the Strava app as a part of extrinsic motivation (ICT motivation), and get insight and feedback on students’ performance through descriptive characteristics of individual training units of the most preferred OASA. The research addressed the following questions:

- What was the students’ motivation to engage in outdoor aerobic sport activities (OASA) in PE?
- What features and tools of the Strava app enhanced the students’ personal ICT motivation?
- Which activities were the most preferred by OASA management?
- What were the training characteristics of students’ sport performance in the most preferred OASA?

## 2 Materials and methods

### 2.1 Research design

The study uses quantitative empirical approach with a cross-sectional observational research design to analyze the students’ motivational concerns in OASA PE and to measure and describe major training characteristics of OASA activities in PE. The learning methods that were used in OASA management are based on the blended learning model (BLM) that draws on the flex model and the enriched virtual model. The core of BLM lies in the technical support provided via fitness trackers in mobile applications or smartwatches. The fitness trackers provided quantified GPS-measured data for students’ self-monitoring and for teachers’ control and analysis to give feedback on training efficiency. The research project was to expand the possible applications of the designed BLM of Chaloupský et al. (2020) to cover a wider range of outdoor sport activities, not only running but also cycling, walking, inline skating, ice skating, cross-country skiing, or ski touring. Coincidentally, quite unplanned, the applicability of BLM itself proved to be a convenient alternative to PE lessons in the time of the COVID-19 pandemic. In the time of distant learning, the BLM was ready to be applied in the PE lessons. This helped the students to stay active and keep fit. In usual situation (no-COVID), the students of FIM UHK can choose from various PE sports activities such as sport games (e.g., volleyball, football, squash, badminton, table tennis), group exercise (e.g., pilates, interval training, yoga, health gymnastics, DNS training—dynamic neuromuscular stabilization), exercise in the gym, indoor climbing, or outdoor sports courses (in winter—skiing, in summer—hiking, or sports by the sea—kayaking, snorkeling, paddleboarding, windsurfing). The PE content in the summer semester of 2021 was organized as outdoor aerobic sport activities (OASA). The OASA was managed in the BLM with support of wearables and the Strava app. The research was carried out to evaluate OASA management using wearables and their potential to address intrinsic and ICT motivation to enhance the university PE learning process. The study describes the management, structure, and organization of OASA as well as the descriptive characteristics of the PE lessons/training units (*n* = 3,680). It is important to emphasize that COVID-19 was not the focus of interest, but provided an unplanned, “unique” opportunity to include those students who would have otherwise chosen a different sport in the usual situation (no-COVID), and thus find out how motivation really worked concerning beginners. The distribution of students’ preferences in no-COVID would have been as follows: sports games (25%), running or cycling (22%), group exercise (22%), individual training in the gym (19%), indoor climbing (7%), and outdoor sports courses (7%).

### 2.2 Research sample

In total, 368 university students (185 women and 183 men, aged 19–23) participated in the empirical research. All participants were students enrolled in the optional subject of PE at FIM UHK, Czech Republic, in the summer semester of 2021. A new sport club called “FIM sport activity” was created on the Strava online platform, and all the students registered and joined the online sport community. The students who passed all the assignments by the end of the semester were included in the research sample. The criteria were: regularity of PA, recording and sharing the training data via the Strava app, filling-in the questionnaire into motivation and ICT motivation in the final evaluation, and 10 training units with the minimum required distance or time limit (see Section 3.1 for details of the training parameters). The research sample was described in terms of previous experience with fitness tracking app (39% yes—some experience, 61% no experience), sport activity in no-COVID time (see Section 2.1 above), route preference (53% road, 47% off-road), and initial motive for participation (25% “I want to exercise,” 75% “I need to exercise” to pass and gain credit).

### 2.3 Data collection, procedures, and analysis

The main technique that was used to collect the data concerning students’ motivation was a structured questionnaire distributed to the participants as a part of their final evaluation at the end of the monitored semesters. The questionnaire provided students’ self-reported data (*n* = 368). Dimensions focused on intrinsic motivation of the students and extrinsic ICT motivation, based on the evaluation of the features and tools of the Strava app. The four main categories of running motivation correspond to the MOMS, Motivations of Marathoners Scale (Masters et al., 1993). The categories include physical, psychological, performance-related, and social motivations. Each category contains three items within the given concern: staying in shape, staying healthy, controlling weight (physical), having fun, improving state of mind, reducing stress (psychological), achieving a goal, overcoming a personal challenge, competing with other runners (performance), self-esteem, gaining respect from friends, and socializing with a running community (social). The data were evaluated in a quantitative analysis using descriptive statistics frequency tables with relative frequency of responses, according to the selected categories of intrinsic motivation and according to the features and functions concerning ICT motivation. The main technique for the training data measurement was fitness tracking via the Strava app. That provided quantified data about distance and time based on real-time GPS measures. The training data were monitored and shared via the Strava online platform and analyzed in descriptive statistics by the application of IBM SPSS Statistics v. 29.0.1.0.

Students could combine outdoor aerobic sport activities in any way according to their preferences: running, cycling, walking, skating (inline skating or ice skating), and skiing (cross-country or ski touring). A total of 368 students participated in the research study and trained regularly 1× or 2× a week over the period of one study semester. Each student had to complete 10 training units in order to pass the final assignment of PE and receive credits. That is why the total number of training units recorded by all the students during the semester was 3,680. In continuous assessment during the whole semester, the PE teachers checked the validity and technical parameters of the students’ records via their training profiles and checked if the selfie photos made during the activity matched the location on the satellite map to approve the Strava records. There were four PE teachers, each of them working with about 90 students during the semester. The PE teachers know most of the students personally, as they meet in sports courses and classes during all semesters of all years of their study.

Descriptive training parameters of the most preferred OASA (running, cycling, and walking) over the course of the semester (Training 1–Training 10) were obtained to provide feedback and insight into sport performance characteristics. Shapiro–Wilk test showed that the data do not have normal distribution. Data in the individual training units for different activities did not seem to be symmetrically distributed based on the frequency histogram, the data are heterogeneous. The analysis in descriptive statistics includes the identification of outliers (min, max) and the representation of data dispersion by median and Q1–Q3 quartiles. Sorting the results into tables describing distribution among individual activities and training units suggests an appropriate point for data interpretation, because the groups were too heterogeneous, with mixed cases among the trainings and activities.

## 3 Results

### 3.1 Use of the Strava app for outdoor aerobic sport activities in PE

**Figure 1:**
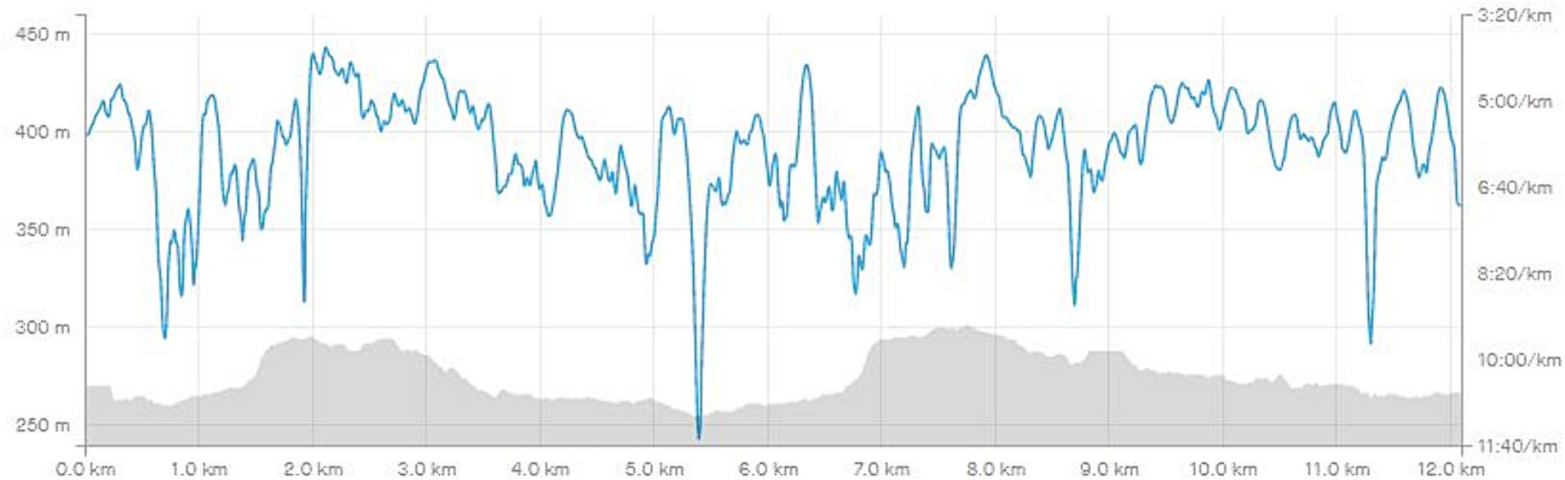
An example analysis of elevation and pace (Source: author’s own records from the profile on Strava).

The core of the blended learning model lies in the technical support, which is proceeded via fitness tracking apps and wearables. After testing and using fitness tracking applications, the Strava app was selected for OASA management in PE of university students. This application is free, easy to use on any smartphone, and allows recording of various physical activities and their subsequent display, reviewing, and browsing. The Strava app was launched in 2009 and is currently used by more than 100 million athletes in 195 countries around the world (Strava, 2023). The Strava app can run on mobile devices with Android, iOS, and also on website platforms. The application enables recording of more than 30 different sporting activities, with the distance displayed on the map base and a graph showing altitude meters and pace. Basic metrics include distance and time, elevation gain, pace, max/min speed, power, energy expenditure, and more. An example of the analysis of a 12.09 km run with a focus on altitude and pace can be found in Figure 1.

**Figure 2:**
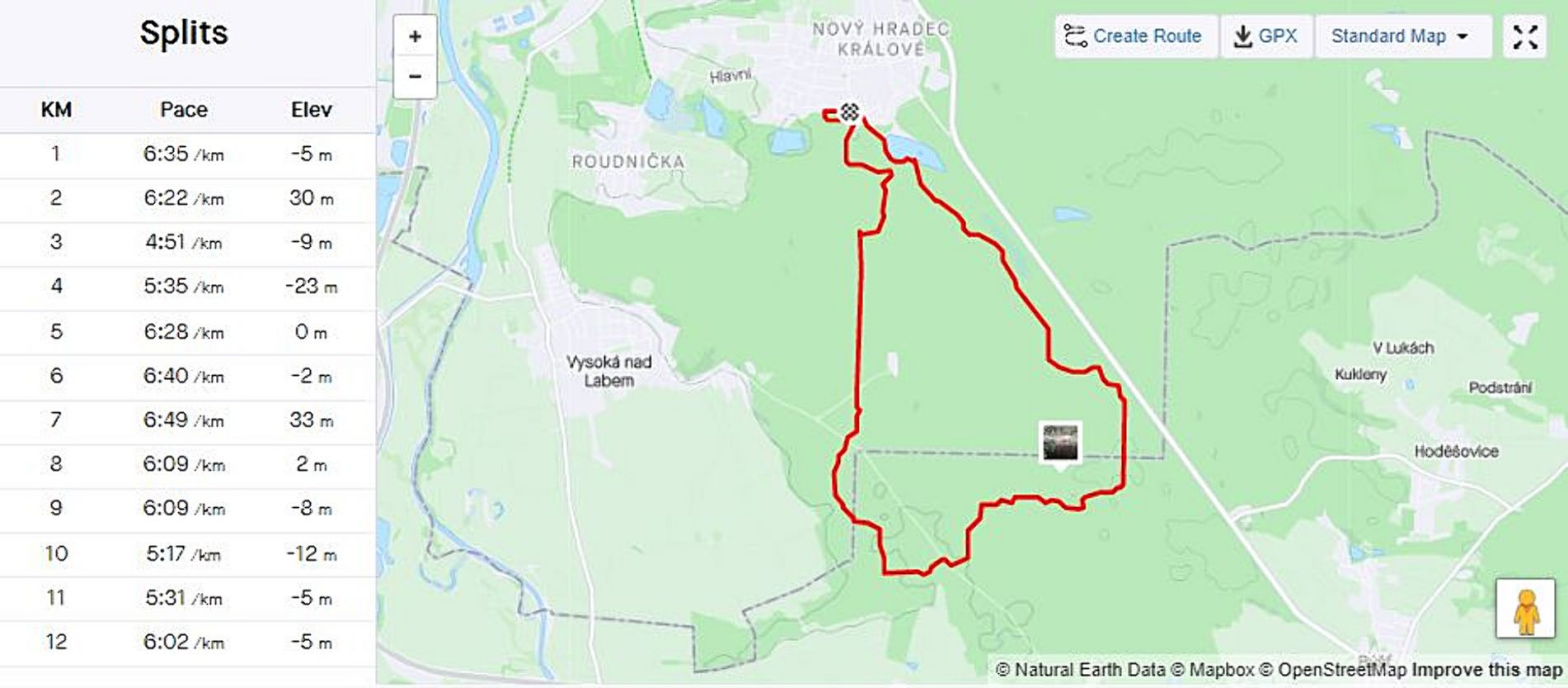
An example of the track record in the map (Source: authors’ own records from the profile on Strava).

Photos can be added to each record either during the activity or afterwards. Then you can clearly see at which point of the training the photo was taken, as in the example of the track record in the map in Figure 2, where you can also see the analysis of the achieved pace in individual kilometers of the distance.

**Figure 3:**
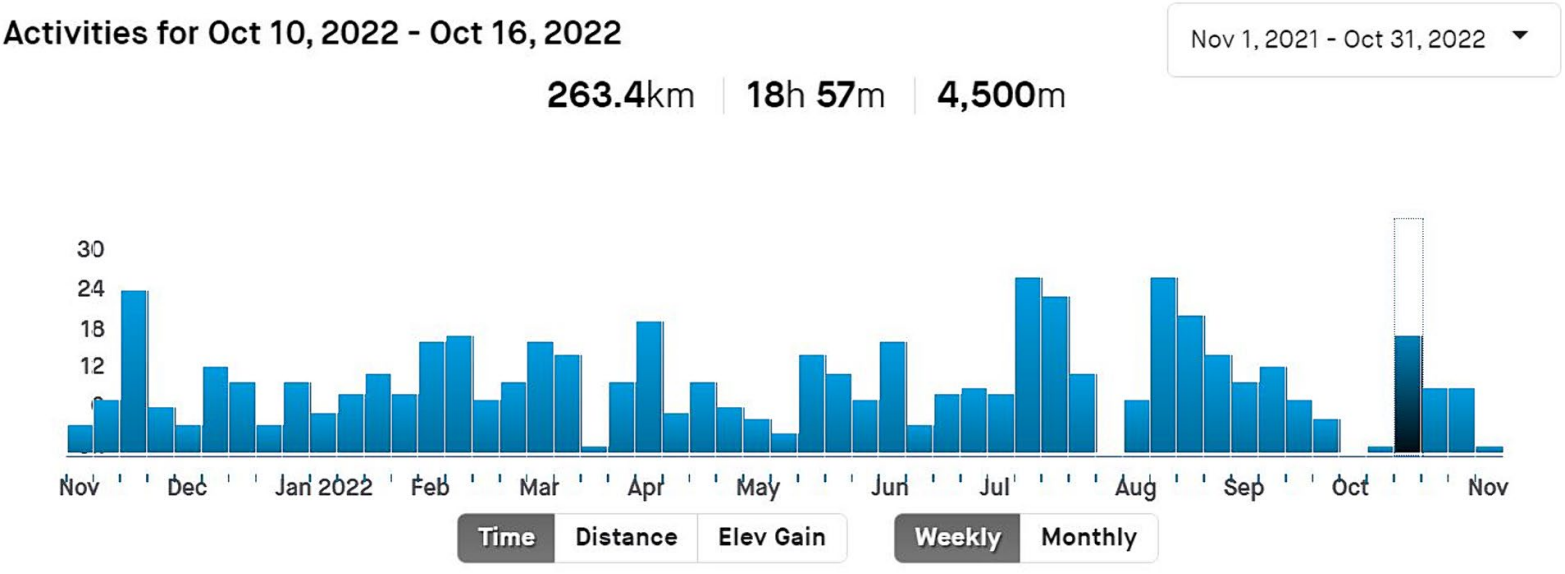
An example training calendar with weekly activity distribution (Source: authors’ own records from the profile on Strava).

A mobile phone with a GPS signal or smart sport watch can be used to record the activity. Most often, the athletes use a smart sport watch, which is easy to pair with the Strava app, and the recorded data will be transferred automatically and then displayed on the Strava platform. An important factor for students of FIM UHK is that they can download the Strava app for free. The Strava app is often referred to as the “athlete social network.” Motivating functions and features of the Strava app include searching for friends, rivals, inspiring athletes, or colleagues from the club. A club of FIM OASA was created for the purpose of managing the OASA of FIM UHK students who enrolled to optional PE. Individual records or profiles can be shared with the whole Strava user community, just with the selected users (followers), or shared through other social networks. In addition to the specific descriptive data that are displayed for each record, you can also monitor the statistical values of training sessions over a longer period of time. It is a so-called training calendar, where training days, hours, kilometers, and personal records are displayed both in numerical data and in the form of graphs. Figure 3 shows an example of a training calendar with distribution of weekly sport activities throughout the year. The numeric values given at the top stand for the total distance (here 263.4 km), time (here 18 h 57 m), and elevation (here 4,500 m) gained in the selected week.

The application also allows one to compare one’s performance with that of other users. The comparison is made through the function of “segments.” Each user on the Strava platform can define their segment. When an athlete completes this pre-defined segment, their performance (time/pace) on the segment can be compared with that of other athletes. The fastest athlete in each segment wins the so-called “virtual crown.” This feature can help motivate maximum personal performance. Another motivation element is the possibility of obtaining a virtual title of “local legend.” This is awarded to the athlete who has completed the selected segment the most times in the last 90 days. Popular motivating tool of the Strava app are “challenges,” which are listed every month. Challenges are, for example, in the form of number of kilometers run or ridden, number of meters climbed in elevation gain, or the duration of sport activity done. If an athlete fulfills the requirement, an icon of completed challenge will appear in their profile in the so-called “trophy case.”

### 3.2 OASA management—structure and the required training unit parameters

The designed structure of OASA is not only about performance but, above all, about motivating students to engage in targeted and regular physical activity. Students shall record at least 10 training units in the Strava app to pass the optional PE course and get credits in each within their whole study, for a maximum of 10 semesters. This provides a longer-term sport training effect. Each PE training unit report must include the track record in the map background, distance, time, and two selfie photos from the terrain taken during each training. The minimum required training parameters for the individual OASA are summarized in Table 1.

**Table 1:** Training parameters.

OASA	Training unit parameters	
Running or 45 min of continuous running	Women: 5 km	
	Men: 6 km	
	60 min of alternate running and walking (option for beginners)	
Cycling or 90 min of continuous cycling	25 km	
Walking	15 km long continuous walking/hiking trip	
	Counted as “double activity”—covers two training units	
In-line skating or ice skating	10 km of continuous activity	
Cross-country skiing or ski touring	10 km of continuous activity	

**Figure 4:**
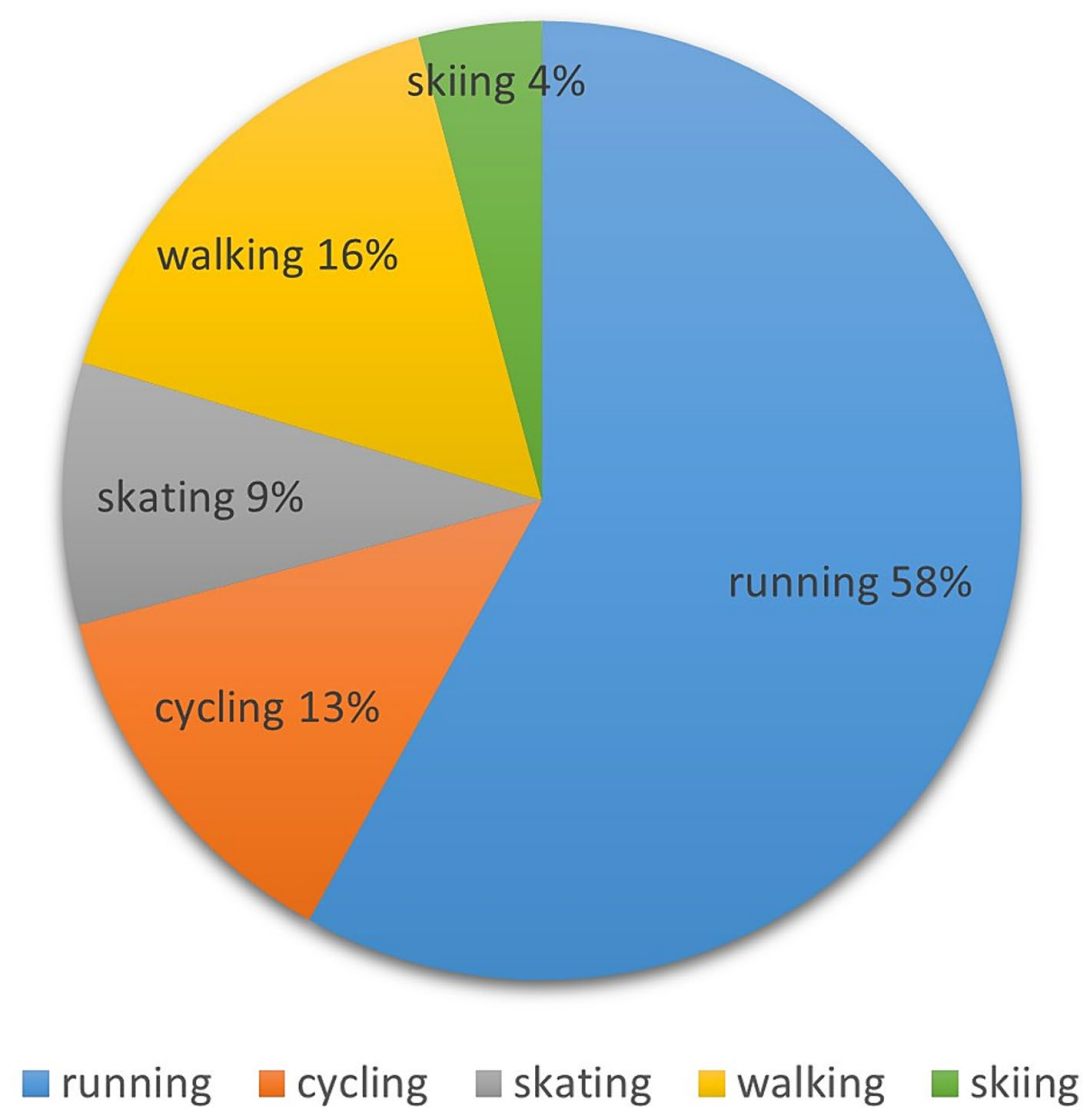
Students’ preferences for different activities.

The training parameters are specified in such a way that they support personalized learning. Running beginners and students whose health or lack of fitness does not allow for continuous running can alternate between running and walking and do a less intense but longer-lasting PA of at least 60 min. Consultation with the PE teacher is required here. A very important factor in sports activity is its regularity for longer-term effects. The frequency of training sessions is 1–2× a week. The study follows the question about the students’ most preferred activities. The distribution indicates that students mainly preferred running, cycling, and walking, as shown in Figure 4. The results show the distribution of each outdoor aerobic sport activity within the total number of training units (*n* = 3,680) performed by all students throughout the semester. Running was the most popular with a total of 2,137 training units (58%), followed by cycling with a total of 469 training units (13%) and walking with a total of 298 training units (16%), where walking was counted as a “double activity”—see Table 1. Skiing had the lowest relative frequency (4%) and skating was not so frequent (9%) within the range of all the given outdoor aerobic sport activities.

An OASA management chart (see Table 2) visualizes and gives a simple summary of OASA management. It helps to effectively organize PE for university students and support their motivation to engage in regular physical activity. The chart gives an insight into practical aspects of the pedagogical process. The designed model follows both theoretical and practical goals that have been described as formal (PE in study curriculum), educational (knowledge acquisition in the field of sport training theory), health (motor abilities and skills, fitness level, well-being), motivational (PA engagement, regularity), diagnostic (input, continuous, and output training assessment), and preparatory (lifelong PA).

**Table 2:** OASA management visualized chart.

Before you start the sport activities
Attend introductory meeting with the teacher—presentation/information (face-to-face or online)
Download Strava app
Choose mobile/smart watch
Create a user account/profile
Upload a profile photography and join the club of “FIM OASA” in the Strava app
Open your profile—activities visible to all members
Test the application in practice
Training guidelines
Set personal goals (consult with the teacher if needed)—get motivated
Choose your preferred OASA—feel free to combine (try, search for the best to fit your individual interests, enjoy)
Be consistent—frequency (1–2×/week), load/intensity—individualize, but follow the minimum performance guidelines (feel free to do more; maximum is not given)
Formal requirements
First training report on time—approved by the teacher
Two photos made in the terrain during each training and recorded to the track record on the map
Minimum 10 reports/training within the semester
Final evaluation questionnaire, feedback, credit assignment
Recommendations
Communicate with the teacher
Stay “connected” through your club FIM OASA (follow others, challenges, kudos, try to get better/push your limits, share your feelings and emotions)
Monitor your performance, check your progress, know your training parameters—running pace in different terrains, subjective feeling in different zones of intensity
Bad weather should not stop you—dress accordingly
Preferably choose trails in nature, explore and discover interesting places

**Figure 5:**
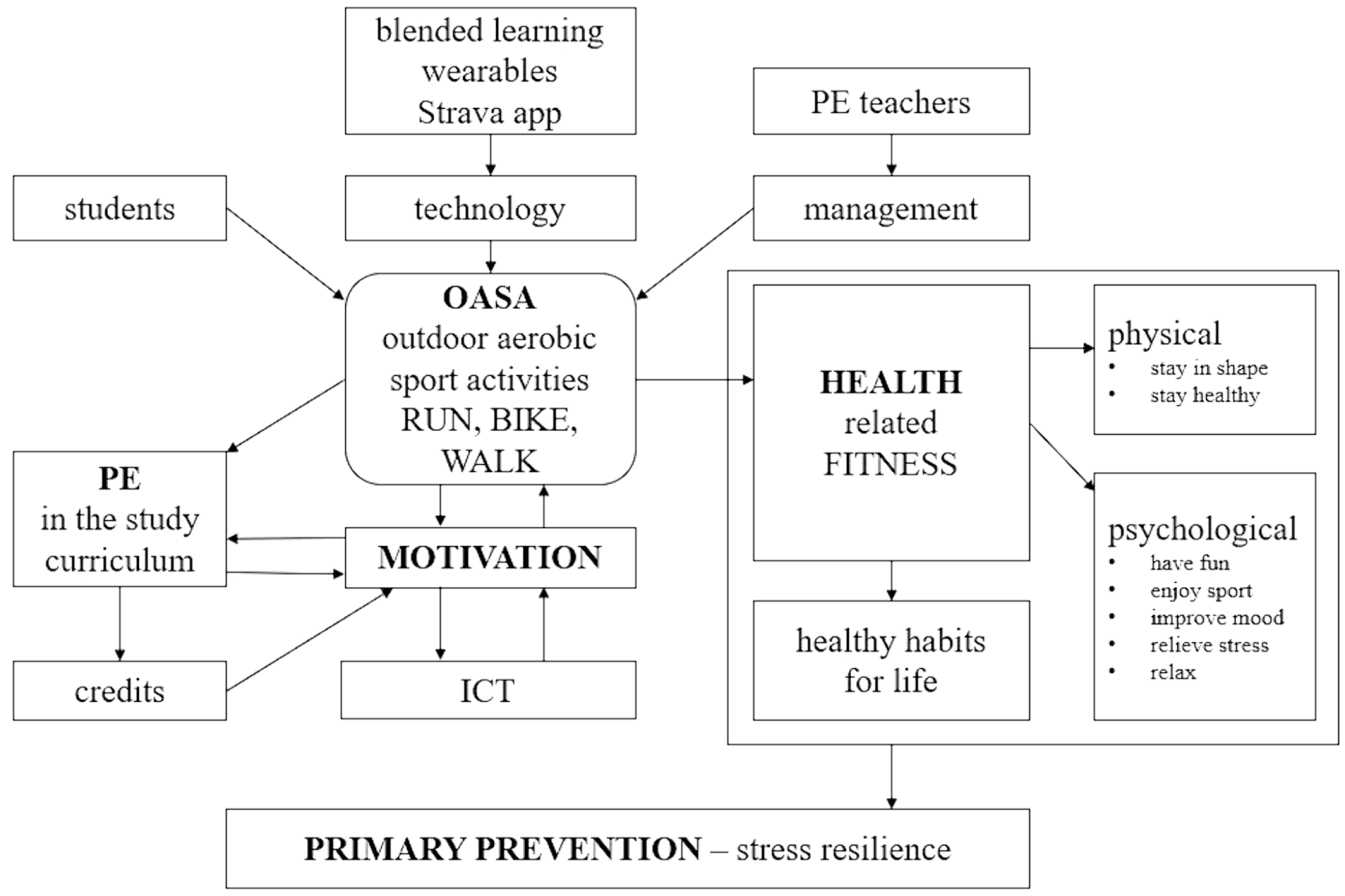
Flow model of OASA management for university PE.

Figure 5 shows a simplified graphical overview of teaching methods, interactions, and relations of individual variables for essential understanding of the system’s educational processes in the OASA management structure. Personalized learning with teacher support is important in the process. The role of the teacher can be divided into three phases, as was structured in the previous study (Chaloupský et al., 2020): initial, continuous, and final. Initial phase contains a joint introductory session, offered both face-to-face and online, students can choose what suits them better. Teacher explains the technical and motivational issues, introduces different types of sport trainings, gives examples of goal setting, recommends individual methods to achieve goals, corrects disproportionate goals helps students with possible technical difficulties and novelty effects of the Strava app in beginner users, and gives support to start. After the student sends his or her first report, at the beginning of the semester, the teacher will check the validity and technical parameters and also if the selfie photos taken during the activity match the location on the satellite map to approve the Strava record. The second phase runs continuously throughout the whole semester, when students train individually and submit their reports electronically via direct links to their personal Strava profile. If needed, the teacher gives comments or recommendations or solves discrepancies by email or invites students for a personal consultation and gives continuous support. The final phase contains the final evaluation of the effectiveness and individual benefits of the whole training cycle at the end of the semester PE course. The final evaluation is recommended as a face-to-face one-to-one session, when the teacher works not only with the performance parameters from Strava records, but also individual goal achievement, discusses students’ feelings and motivation to personalize the training and gives support to carry on in regular PA.

### 3.3 Students’ motivation

The analysis of the questionnaire responses showed the intrinsic motivation of the students to engage in physical activity. The results were processed in terms of the relative frequency of individual motivations within the given four categories of concerns (physical, psychological, achievement, and social). The results in Table 3 are arranged in descending order, from the motivations that apply to the largest percentage of students to the motivations that do not motivate students that much.

**Table 3:** Students’ motivation.

Students’ motivation to do OASA	Concern	%
Stay in shape	Physical	94
Stay healthy	Physical	90
Have fun/enjoy the activity	Psychological	88
Improve state of mind/mood	Psychological	88
Relieve stress/relax	Psychological	83
Self-esteem/self-respect	Social	82
Achieve a goal	Achievement	80
Meet a personal challenge	Achievement	72
Control weight	Physical	57
Socialize with a running community	Social	54
Respect of friends/family	Social	48
Compete with other runners	Achievement	32

The most common motivations included physical concerns to stay in shape (94%) and to stay healthy (90%). Psychological concerns were also generally rated high when it comes to how many students consider OASA as a means to have fun (88%), improve their state of mind (88%), or relieve stress (83%). For achievement concerns, the results show that the focus is more on a personal challenge (72%), while competing with others was ranked lowest (32%).

### 3.4 ICT motivation

Two findings emerged clearly from the analysis of ICT motivation in Table 4. First, students were motivated by the possibility of monitoring and analyzing their own individual training parameters (60% in terms of distance, 61% in terms of speed or pace, 57% in terms of viewing and browsing individual records as such, and 70% in terms of comparing overall performance within their individual training units). Secondly, most students did not compare their own performance with that of other athletes. These results are consistent with the non-competitive focus of OASA within the PE classes. 57% of participants enjoy listening to music during a sport activity to motivate themselves to exercise. The use of GPS for navigation is appreciated and used by 43% of the participants. At the same time, 34% of participants usually choose the same route, so they simply do not need navigation. Nevertheless, some attempts (40%) to discover new places can be seen. Here, the preferences of individual students can be traced in the records of their routes during sport. The Strava app suited the majority of students (78%).

**Table 4:** ICT motivation.

Students ICT motivation	Agree	Indifferent	Not agree
Via application Strava app	*n* (%)	*n* (%)	*n* (%)
Browsing and watching own records	210 (57)	93 (25)	65 (18)
Watching the distance within individual training	221 (60)	66 (18)	81 (22)
Watching the speed or tempo within individual training	225 (61)	68 (18)	75 (20)
Comparing the performance of individual training	258 (70)	50 (14)	60 (16)
Following other members in the FIM OASA club	112 (30)	80 (22)	176 (48)
Comparing own performance with other members of STRAVA	82 (22)	60 (16)	226 (62)
Evaluation or comments on my performance by others (kudos/likes)	73 (20)	55 (15)	240 (65)
Following the records within the FIM OASA club	65 (18)	63 (17)	240 (65)
Various challenges that are on STRAVA (e.g., to run 10 km)	61 (17)	66 (18)	241 (65)
Listening to music in my headphones/air pods	211 (57)	47 (13)	110 (30)
Using GPS not only for recording but also for navigation on the track	158 (43)	45 (12)	165 (45)
Choosing the same track all the time	124 (34)	119 (32)	125 (34)
Trying to discover new places when planning the track	149 (40)	113 (31)	106 (29)
Strava app is suitable for recording of individual training	287 (78)	49 (13)	32 (8)

### 3.5 Training performance—running, cycling, and walking

The Q1 and Q3 quartiles and the outliers descriptively characterize both ends of the data distribution. The data are shown separately for each group: running, cycling, and walking, for both men and women. The data were used for analysis to uncover and find different aspects and relationships and to examine the distribution of the data. The interquartile range contains 50% of the values of the measured distance and is divided into two parts by the median. If the median is close to the value of one of the quartiles, this indicates that the data are skewed in the opposite direction.

The minimum number of kilometers to be run was set at 6 km for men (Table 5) and 5 km for women (Table 6). The distance run by the students in the majority of the training units oscillated around this minimum limit. In parallel, there was the option of a time limit of at least 45 min of continuous running for the runners. This second option was mainly used by beginners who were not able to run at a pace that allowed them to run 6 km in <45 min. In this case, the distance was shorter than the required kilometers. The distance covered during the running training for most men was between 6.0 and 7.3 km, in the range Q1–Q3. For the women, it was 5.0–6.5 km. The maximum individual values for the individual training units (training 01–10) were 12.0–15.2 km for the men and 10.0–12.0 km for the women. The mean tendency of the distribution of the values (median) is closer to the values of Q1. It is 6.2–6.4 km for men and 5.2–5.4 km for women. Most men and women stopped their training and exited GPS and the Strava app shortly after reaching the minimum required distance limit (described in the assignment parameters).

**Table 5:** Descriptive statistics of running training units—men.

Running (km)	Men					
	*n*	Min	Q1	Median	Q3	Max
Training 01	103	5.0	6.0	6.3	7.1	14.3
Training 02	104	4.8	6.0	6.2	7.2	14.6
Training 03	101	4.4	6.1	6.3	7.0	13.5
Training 04	106	5.1	6.1	6.4	7.1	12.0
Training 05	103	4.6	6.0	6.3	7.1	13.1
Training 06	94	5.0	6.0	6.3	7.0	13.1
Training 07	91	5.2	6.0	6.3	7.3	13.6
Training 08	87	5.0	6.1	6.3	7.1	12.0
Training 09	95	5.0	6.0	6.2	7.0	12.3
Training 10	91	5.2	6.0	6.3	7.1	15.2

**Table 6:** Descriptive statistics of running training units—women.

Running (km)	Women					
	*n*	Min	Q1	Median	Q3	Max
Training 01	122	5.0	5.1	5.4	6.0	11.5
Training 02	121	5.0	5.0	5.2	6.0	11.1
Training 03	133	4.9	5.1	5.3	6.1	11.4
Training 04	121	5.0	5.1	5.3	6.0	10.2
Training 05	118	5.0	5.1	5.3	6.2	10.0
Training 06	115	4.2	5.1	5.4	6.0	10.5
Training 07	110	5.0	5.1	5.4	6.1	11.5
Training 08	111	4.1	5.0	5.2	6.0	11.1
Training 09	105	5.0	5.1	5.3	6.0	11.7
Training 10	106	5.0	5.1	5.4	6.5	12.0

The lower limit of cycling kilometers was set at 25 km for both men and women. The descriptive characteristics can be found in Table 7 for men and Table 8 for women. This distance limit was chosen by the majority of cyclists. In parallel, there was the option of a time limit of 90 min of continuous cycling. This second option was used by beginners who were not used to cycling for such a long time or at a given pace. The time limit was also used by students who preferred mountain biking on challenging and hilly terrain. Therefore, some values are lower than 25 km. The tendency of the mean distribution of the values (median) varied in the individual training units, as shown in Tables 7, 8.

**Table 7:** Descriptive statistics of cycling training units—men.

Cycling (km)	Men					
	*n*	Min	Q1	Median	Q3	Max
Training 01	36	19.9	25.1	26.7	29.5	90.5
Training 02	31	19.9	26.3	27.7	31.5	36.2
Training 03	40	20.4	25.9	29.4	31.1	62.8
Training 04	36	24.4	26.4	28.8	38.2	74.7
Training 05	33	23.2	25.8	28.4	38.9	57.1
Training 06	38	16.4	25.8	29.6	34.5	68.8
Training 07	39	16.6	25.6	28.7	32.2	98.8
Training 08	40	17.5	26.6	28.2	33.2	67.7
Training 09	40	16.7	25.8	28.6	35.4	70.3
Training 10	41	23.0	25.5	27.8	35.6	74.6

**Table 8:** Descriptive statistics of cycling training units—women.

Cycling (km)	Women					
	*n*	Min	Q1	Median	Q3	Max
Training 01	7	20.0	21.7	24.1	25.7	26.4
Training 02	8	20.8	24.9	27.9	29.9	31.2
Training 03	7	25.0	25.2	29.4	35.1	37.3
Training 04	8	19.6	20.9	26.0	27.7	32.6
Training 05	15	16.9	21.8	25.2	37.3	50.0
Training 06	14	19.1	21.4	26.0	29.4	45.7
Training 07	13	12.3	20.0	25.8	29.0	42.7
Training 08	13	22.9	25.5	26.0	31.2	35.5
Training 09	18	17.9	23.1	25.9	29.0	38.1
Training 10	19	15.8	24.0	26.0	31.2	60.2

The minimum limit for walking kilometers was given as 15 km per one training unit (or better called “walking/hiking tour”) for men and women. No time limit was set, but walking was clearly more time-consuming compared to running or cycling. Therefore, walking was counted as a double training unit. Therefore, only five training sessions are listed in Table 9 for men and Table 10 for women. The distance covered while walking varied for most training units, in the range of Q1–Q3 between 15.1 and 24.3 km for men and 15.2–18.7 km for women. The maximum individual values for each training session (training 01–10) were 19.6–32.8 km for men and 22.0–25.6 km for women. The tendency of the mean distribution of values (median) shifted a little closer to the values of Q1. Most students ended their training shortly after reaching the limit of 15 km.

**Table 9:** Descriptive statistics of walking training units—men.

Walking	Men					
	*n*	Min	Q1	Median	Q3	Max
Training 01	44	14.8	15.1	15.6	17.1	27.2
Training 02	43	14.9	15.1	15.5	16.8	22.3
Training 03	38	15.0	15.2	16.0	17.7	32.8
Training 04	19	15.0	15.3	15.6	17.8	19.6
Training 05	10	15.0	15.6	16.4	24.3	29.0

**Table 10:** Descriptive statistics of walking training units—women.

Walking	Women					
	*n*	Min	Q1	Median	Q3	Max
Training 01	36	15.0	15.3	15.9	17.6	25.6
Training 02	38	15.0	15.3	16.4	18.3	22.0
Training 03	28	15.0	15.2	16.0	18.3	25.6
Training 04	24	15.0	15.3	16.4	18.7	23.7
Training 05	18	15.0	15.6	17.4	17.9	23.1

## 4 Discussion

The aim of this study was to evaluate outdoor aerobic sport activities (OASA) in physical education (PE) of university students, using wearables with the Strava app and their potential to effectively promote learning process, personalize methods, and enhance motivation in a blended learning approach. The used blended learning model (BLM) was designed and verified in detail in the previous research (Chaloupský et al., 2020). The designed OASA management structure was evaluated with a focus on the findings of current relevant studies discussing recommendations about content and methods in the context of university PE. It can be seen that OASA management in BLM complies with the need to search for solution of decline in physical activity in university studies (Bebeley et al., 2018; McFadden, 2021) and adolescence (Ahmad et al., 2021; Kemp et al., 2021), decline in physical fitness and motor abilities (Kaj et al., 2015; O’Brien et al., 2022), increase in sedentary behavior and screen time (Thievel et al., 2021; Gut et al., 2022), and possible increase in the percentage of total body fat (Šipl and Hurych, 2022).

Integration of PE into university studies, to which our study appeals, is a discussed topic that can be empirically justified through findings from current research studies (Bertocchi et al., 2021; Podstawski et al., 2021; Shi, 2023). The question is how to motivate university students to pursue PA. The period of transition from adolescence to young adult age, i.e., from high school to college or universities, seems to bring a risk of decreased PA and motivation and change in exercise behavior (Podstawski et al., 2021), which has also been observed at the University of Hradec Králové (UHK). A decrease in moderate-to-vigorous PA among university students, following the transition from high school, was also confirmed in the study of Wilson et al. (2022), emphasizing the importance of addressing both intrinsic and extrinsic motivators. Following this line, the results of our study provided an optimized structure of OASA management that can be effectively applied in college and university PE, supporting students’ engagement in regular physical activity and their self-determined motivation through the individualized guidance of the teacher and a fitness tracking app.

At the starting point of the study, major factors were identified at UHK that may have been decreasing students’ engagement in PE due to their daily study schedule and routines, motivation, but also a lack of PE teachers’ capacity and sport facilities. The factors are similar to those of the systematic review of Silva et al. (2022), identifying the main barriers to PA in lack of time, lack of motivation, and lack of accessible places. Similar problems were identified in the research of 66 other European tertiary institutions (Podstawski et al., 2021). The results indicate that the importance of PE has been steadily declining in higher education in recent decades since more than 30% of the analyzed institutions had their PE curricula reduced to a minimum or removed from the study programs (Podstawski et al., 2021). That has also been a case at UHK, where only one faculty (the Faculty of Informatics and Management [FIM]), out of four, has PE as a part of the study curriculum. The OASA management structure, with the use of fitness trackers in BLM, suggests a partial possible solution to the discussed problem. This approach can be supported by the findings of more studies concerning fitness tracking, PA, and motivation (Gowin et al., 2015; Chaloupský et al., 2020; Ahmed and Leung, 2021; Mokmin and Jamiat, 2021; Smith and Volkwyn, 2022).

OASA management seems to work effectively on the basis of optional PE courses, which are elective parts of the study curriculum. A similar approach is suggested in the study of Kim and Cardinal (2019) examining the psychological and behavioral characteristics of freshman students, where the main finding was that students felt more competent and motivated in elective courses compared to compulsory conditions. This is in agreement with Deci and Ryan (2000), who explain that the obligation to take a course may be met with resistance.

It can be seen that the students generally appreciated the opportunity to connect with the sport online community at FIM OASA and belong somewhere to share their experiences. The distribution among the total of the recorded OASA training units showed the preference of activities: running was the most popular (58%), followed by cycling (13%) and walking (16%). The results can be compared to the study examining leisure-time PA at the Maryville College faculty, Steeves et al. (2023), when staff’s primary outdoor activities were walking (47%) and running (28%), students preferred walking (35%), and other sport (33%), while both groups reported a preference for group outdoor activities (82%). Similarly, running, walking, and cycling are part of the current trend of escapism while staying connected through screens and social media (Helsen et al., 2022). Concerning the choice of sport activities, outdoor sports are considered appropriate for adolescents and young adults (Tammelin et al., 2003; Blond et al., 2016). Concerning the intensity of PA, aerobic activities proved to be the more popular type in comparison to anaerobic activity among university students in the study of Smith and Volkwyn (2022). However, it should be highlighted that there may be different needs between those with preferences for individual and team sports to enhance PA level, as concluded in the study of Ruth et al. (2022). Only a few studies were found with a primary focus on outdoor sport aerobic activities in university PE. The study of Steeves et al. (2023) examined leisure-time outdoor behaviors of college students and faculty employees and found the top three perceived health benefits of spending time outdoors were improved health, overall health, and enjoyment of life. Outdoor sport activities are generally indicated to provide health benefits related to simply being outdoors, such as improvements in physical health, mental health, well-being, education, lifelong learning, or other social benefits (Eigenschenk et al., 2019). Outdoor sport activities can also have multiple health benefits, such as preventive effects against vitamin D deficiency, multiple sclerosis, or osteoporosis (Manferdelli et al., 2019). A research gap was revealed, especially in the long-term effects that outdoor sports could have on personal development (Eigenschenk et al., 2019), concerning recommendations to adopt lifelong PA. It is suggested that a longitudinal study be conducted at FIM UHK in the future to record and monitor the PA and motivation indicators in subsequent years to obtain statistically significant results.

The analysis of the research results in students’ motivation suggests several relationships concerning the intrinsic and the extrinsic ICT motivation for PA. The results showed that students were most motivated to participate in OASA by physical concerns of staying in shape (94%), and staying healthy (90%). Similar results in health-related factors were obtained in the study of runners by Rozmiarek et al. (2022). Hewitt et al. (2022) agree that health motives can be a valuable tool because individuals who prioritize their health as a motive for exercise tend to be more active. The physical concerns were followed by psychological concerns of motivation in terms of enjoyment (88%), improving mood (88%), or stress relief (83%). Another study examined the effectiveness of app-based intervention in a mixed approach among university students and found that it may affect students’ enjoyment, in addition to self-efficacy and family support (Melton et al., 2015). The increase in enjoyment of life was also indicated both in students and staff at Maryville College, based on their participation in outdoor sport activities (Steeves et al., 2023). Sport activities are undoubtedly associated with achieving personal goals, comparing one’s performance, and overcoming oneself. In the results of achievement concerns in intrinsic motivation questionnaire, competing with other runners was the least influential motivator (32%), while achieving a personal goal had a high value (80%). This finding can be explained by the self-regulatory approach (Ryan and Deci, 2020) and is correlated to the ICT motivation questionnaire results, where students preferred competition to outperform themselves (70%) as a personal challenge, while the opportunity to compare with other athletes on Strava was valued as a low motivator (22%). Positive aspects of personal goal achievement with the help of fitness trackers are discussed to increase motivation and PA engagement in other studies (Ramirez and Bernhardt, 2020; Kendrick et al., 2024). It is suggested that providing opportunities that are non-competitive may help university students to adopt a long-life sport-for-all orientation, with the potential to increase long-term PA and a healthy lifestyle in general (Pheiffer and Wierenga, 2019).

Social motivational concerns such as gaining the status of a “runner” and recognition of one’s performance by friends or family were important for about half of the research participants. This may be a concern with the potential to be further increased in the field of physical education, as when people have social support, they are more likely to engage in physical activity (Hewitt et al., 2022; Kendrick et al., 2024). A comparison can be made regarding the concern of social connectedness and relatedness, which was rated lower (38%) in the previous research on fitness running with fitness trackers and the Endomondo app (Chaloupský et al., 2019). This suggests that the factor of creating a new online sports club of FIM physical activities on the Strava platform may have played a role in the higher rating of connectedness and relatedness (54%) in the research on OASA management. There is a need to understand the growing popularity and motivational opportunities of online social fitness platforms, such as Strava (Rivers, 2020). However, more factors should be taken into account concerning enhancement of students’ social connectedness, for example, their time spent outdoors, as in the study of Barfield et al. (2021). A recent analysis (Durán-Vinagre et al., 2023) of university students’ motivational processes and intention to be physically active showed statistically significant differences (*p* < 0.01) in favor of men in terms of intrinsic, integrated, identified, and introjected regulation of PA. Men were also more likely to be physically active in the future than women. However, in our study, the comparison between men and women was not the analysis content, as the focus of this research stage was to promote physical activity engagement through appropriate content and methods and verify OASA management in practice. The men/women motivational factors analysis is recommended for the next stage of the research.

Concerning the results on ICT motivation, the majority of our students in OASA activities were motivated by the possibility of monitoring their own progress (70%) between individual training and analyzing and comparing their own individual training parameters in terms of distance (60%), speed, and pace (61%). Recording and saving data for personal use in the mobile fitness apps were rated at a similar level (67%) in students’ leisure activities in the study of Berbeka et al. (2023). This corresponds to the findings of Rapp and Tirabeni (2020) in amateur athletes, who explore and analyze their performance via apps and smart devices as a part of self-motivation. A common function for recording PA is GPS. The results showed that 43% of students used GPS not only for recording but also for route planning and navigation. This correlates to the finding that 40% of students are driven by curiosity to discover new tracks and terrains and can be compared to another study, where GPS was students’ most frequently used app functionality (45%), followed by a training plan (18%) and a training assistant (17%) (Berbeka et al., 2023). Wearable devices are considered superior to mobile phones alone to monitor PA (Bardus et al., 2021); however, our results show that students often start with mobile phone connected to the Strava app after they enroll to OASA PE lessons, and they purchase a smartwatch later, when they gain some training routine. This is considered a desirable step for adopting lifelong PA. At the end of the semester, 84% of students used mobile phone, and only 16% used smartwatch. This can be supported by the finding of Janssen et al. (2020) that people are more likely to use apps when running is their main sport. Users aged 24–29 tend to use smartphones more often compared to users aged 30–49 (Soulé et al., 2021). The highest valued benefit of the OASA management with the Strava app in ICT motivation was the possibility of individual choice of time (91%), place (92%), and when and where to go training. This is confirmatory to the finding that blended learning makes the learning process less limited by time, space, or psychological state (Chao et al., 2021).

On the other hand, in the focus on usability of fitness trackers in general, several possible risks or negative aspects should be discussed, as mentioned in the following studies. The findings of Russell et al. (2023) support the utility of Strava and perceived psychosocial motivation to run among collegiate club runners on the one hand, while on the other hand, they also suggest some potential concerns related to social pressure and self-presentation that could influence mental or physical health. The ups and downs of fitness-related technology are mentioned in the study of Kendrick et al. (2024), with negative aspects like feeling overwhelmed by data or demotivated when goals are not achieved. In the studies of PA in children and adolescents, some of them reported technical difficulties and novelty effects when using wearables (Creaser et al., 2021). A study exploring the use of wearables and fitness apps by student-athletes at the American University of Beirut found that many participants discontinued use due to loss of interest or technical issues (Bardus et al., 2021). Developers of smart fitness tracking devices are advised to provide users with some practical features that can strengthen their perceived usefulness and positive attitude toward technology (Wang et al., 2022). We believe that the above-mentioned negative aspects even highlight the importance of well-structured instructions and teachers’ support in the blended learning model of OASA management. This can also be supported by the study of Hamid et al. (2019), who confirm that the smartphone sport tracker application alone cannot motivate people to perform physical activity. Thus, although fitness tracking apps can seem like a promising innovative tool, it is important to outline an effective strategy and methodological approach, especially in terms of addressing intrinsic motivation.

We did not find other recent studies with a primary focus on training performance parameters in university PE. This gap allows us to speculate on our results and deduce descriptions of students’ exercise behavior. The suggested outcomes are recommended for further research to obtain statistically significant results. In the case of our research study, about half of the participating students (52%) reported that they would have exercised less if they had not participated in OASA. This is considered an important achievement of the OASA management approach. Another great achievement in terms of motivation is the reported finding that 363 out of 368 participating students (99%) would enroll again in the PE courses organized as OASA with the support of the Strava app if they had the opportunity. This suggests that 52% of students who claimed they would have exercised less were properly motivated by the OASA management system. The fact that students need to be motivated to exercise can be confirmed by the analysis of the statistical descriptive parameters of all the recorded training units in the whole semester. There was an obvious tendency for many students to only complete the required minimum number of kilometers and stop recording and exercising exactly when they reached the minimum number of kilometers (or somewhere around the minimum). This corresponds to the finding that only 30% reported they tried to achieve an increase in their training volume or intensity during the semester. This fact underlines the importance of motivating students from the outside, giving teachers’ support, and providing personalized practical guidance in terms of training parameters (recommended activity, volume, frequency). Accordingly, the teacher’s support, interaction, and feedback are considered important in other studies (Ahmed and Leung, 2021; Choi et al., 2021; Smith and Volkwyn, 2022). Recording training sessions via the Strava app motivated students to be active regularly, keep up the pace, or stay in training. “The application got me out the door and forced me to stay in the workout even if it did not feel like working out that day. And I always ended up enjoying it.” Regularity is suggested as an additional benefit for university students. As found in another study, regular running can increase academic performance (Wunsch et al., 2021; Du et al., 2023). Du et al. (2023) examined the minimum average frequency of once a week and the duration of 16–25 min, while OASA management requires once or twice a week and a duration of 30 min or 5 km for women and 30 min or 6 km for men in running. The discussion was about the requirement to take two selfie photos during each training unit. This was reported as a restraint by students, especially those who were more concerned with measuring their time and speed performance, as this interruption may have slightly affected their time and pace data, both in running and cycling. However, in terms of evaluation, photos are an essential tool for teachers in OASA for assessing the originality of individual records.

A systematic review by Nuss et al. (2021) suggests that wearable fitness trackers prove effective in improving motivation for PA in adults; however, studies of college-aged students revealed mixed results. Wallar (2021) conducted a study to understand college students’ motivations for and discontinued use of fitness-related technology, revealing that despite the positive impact of fitness trackers on motivation and behavior, the college students still did not meet the recommended PA, indicating a research gap and a need for establishing practice guidelines for the use of fitness-related technology among college students. Here, the strategy of our research study may help to partly bridge the gap, having designed and verified OASA management as an optimized teaching blended learning method, offering students a practical guideline and tailored support with goal setting, use of the Strava app, and continuous evaluation.

Based on the findings and knowledge gained in our study, most of the recent research studies concerning the use of wearables and smartphone apps to monitor PA are focused into students’ leisure-time PA and not so much into PE. We suggest that further theoretical and scientific solutions should focus on the use of PE in college and university. Despite the general need to promote PA for healthy lifestyle and primary prevention of many substantial health problems, PE has not been an integral part of the study curriculum at some colleges and universities. Here we can find an implication of the focus of our study for society at large, as if students develop a healthy habit of regular PA during their studies, the positive exercise behavior may carry on into adulthood. However, the positive habit and regularity of PA must be established and supported in schools, and this chain must not be broken during the period of students’ transition from high school to college or university. Practical recommendations are to search for solutions that are not complicated, expensive, or demanding to implement. We believe that the OASA management structure may offer a potential solution for further use by experts in PE practice.

## 5 Conclusion

The research brings findings about university students’ motivation for physical activity and implementing blended learning into university PE in the optimized structure of outdoor aerobic sport activities (OASA management). The blended learning model works with the fitness tracker support and Strava app, but highlights the teacher’s role in a continuous process evaluation, giving feedback and support. There are a considerable number of factors that make each student’s self-determined motivation unique. Therefore, personalization of the educational process in university PE can be an effective strategy to respond to students’ individual needs.

Students’ motivation to participate in OASA was found to be mainly related to health and psychological concerns, such as staying in shape, staying healthy, having fun, improving mood, or stress-relieving. The students’ preferred activities were running, cycling, and walking. Among the concerns of ICT motivation, students appreciated the possibility of monitoring and analyzing their records as to distance, time, and pace, and the most valued benefit of the BLM was the opportunity for an individualized choice of time and place. The results showed that most students stopped training in the Strava app as soon as they reached the required minimum kilometers or times. This underlines the need for students to be well motivated to adopt regular physical activity. It is emphasized that PE should be a part of the university study curriculum. OASA management is suggested as an example of an innovative PE method.

### 5.1 Limitations and future research

There are some limitations and future research recommendations for this study that should be considered. It is important to emphasize that COVID-19 was not the main focus of interest. However, it provided an unplanned opportunity to implement the blended learning model of OASA and allowed us to examine its applicability and motivation in a wider research sample. On one hand, it provided a good opportunity to involve students, who, under normal circumstances (“no-covid”), would have chosen different sport activities (indoor activities such as sports games or fitness activities or exercise). On the other hand, the motivation and desire to engage in outdoor sports activities among students may have increased due to the fact that other options for organized sports were decreased by COVID-19 restrictions. Therefore, it is recommended that this be further investigated. In terms of analysis, our results have brought only descriptive statistics because the groups were too heterogeneous, with mixed cases among the training activities. This fact may have been due to the fact that students were free to combine outdoor aerobic sport activities in any way according to their own preferences. However, the focus of this research stage was more into the applicability of the OASA management with BLM into PE university education practice, to individualize the training and personalize learning. It is suggested that a longitudinal study be conducted at FIM UHK in the future to record and monitor the PA and motivation indicators in subsequent years to obtain statistically significant results to examine longer-term PA engagement.

Practical limitations, that may have been caused by the technology itself should also be mentioned here. Flawless functioning may not be guaranteed in all circumstances, and if the technology failed, students had to repeat the activity recording. It concerns the accuracy of GPS signal, dependency on the type of mobile phone or smartwatch, battery life, or the method of data transmission to the application. Another practical limitation may be the requirement to take two selfie photos along the route. However, this is considered a necessary validation measure for the teachers to eliminate the possibility of someone uploading unfair records because the photos can be matched to direct location on the satellite map and the Strava record can be checked and proved manually. The Strava app usage is based on the assumption that it will remain free in the future. If it were to become a paid service, it could be a problem for some students, and it would be necessary to search for alternative technical solutions.

## Data Availability

The raw data supporting the conclusions of this article will be made available by the authors, without undue reservation.
